# Supplementary material for: From sequence to enzyme mechanism using multi-label machine learning
Source: BMC Bioinformatics. 2014 May 19;15:150. doi: 10.1186/1471-2105-15-150 (PMC4229970; doi:10.1186/1471-2105-15-150)
Supplement: Additional file 2 — Java code of ml2db. Additional file ml2db_code.tar.gz contains the Java source code to run the multi-label machine learning experiments and save the results to database. The code’s Javadoc is included. [file 1471-2105-15-150-S2.zip › additional file 2/ml2db/ml2db/doc/main/ml2db/al/utility/confidence/ConfidenceCalculator.html]

ConfidenceCalculator


JavaScript is disabled on your browser.


- Overview
- Package
- Class
- Use
- Tree
- Deprecated
- Index
- Help

- Prev Class
- Next Class

- Frames
- No Frames

- All Classes

- Summary:
- Nested |
- Field |
- Constr |
- Method

- Detail:
- Field |
- Constr |
- Method


main.ml2db.al.utility.confidence

## Class ConfidenceCalculator

- java.lang.Object
- - main.ml2db.al.utility.confidence.ConfidenceCalculator

- ---

    

  ```
  public class ConfidenceCalculator
  extends java.lang.Object
  ```

  Gets the confidence of a multi-label prediction and calculates how close it
  is to 50% (maximum uncertainty)

  Version:
  :   19 March 2012

  Author:
  :   Luna De Ferrari ldf(at)st-andrews.ac.uk

- - ### Constructor Summary

    Constructors

    Constructor and Description

    `ConfidenceCalculator(int storageSize)`
  - ### Method Summary

    Methods

    | Modifier and Type | Method and Description |
    | --- | --- |
    | `static double` | `getAverageUncertainty(double[] confidences)` |
    | `static double[]` | `getConfidences(mulan.classifier.MultiLabelLearner trainedLearner, IdInstance instance)` // input: trained model // actions: get MultiLabelOutput from trained model using // .makePredictions(Instance), // get confidence[] --> one confidence for each class label |
    | `ConfidencesStorage` | `getStore()` |
    | `double` | `getUncertainty(mulan.classifier.MultiLabelLearner trainedLearner, IdInstance instance)` For each confidences set (double[]), calculate the uncertainty and store it. |
    | `static double` | `getUncertaintyValue(double confidence)` // the confidence certainty decreases the closer it is to 0.5 // (e.g. |

    - ### Methods inherited from class java.lang.Object

      `equals, getClass, hashCode, notify, notifyAll, toString, wait, wait, wait`

- - ### Constructor Detail


    - #### ConfidenceCalculator

      ```
      public ConfidenceCalculator(int storageSize)
      ```
  - ### Method Detail


    - #### getStore

      ```
      public ConfidencesStorage getStore()
      ```

      Parameters:
      :   `maxConfidence` - m\_maxConfidence to set


    - #### getUncertainty

      ```
      public double getUncertainty(mulan.classifier.MultiLabelLearner trainedLearner,
                          IdInstance instance)
      ```

      For each confidences set (double[]), calculate the uncertainty and store
      it.


    - #### getAverageUncertainty

      ```
      public static double getAverageUncertainty(double[] confidences)
      ```

      Parameters:
      :   `confidences` -

      Returns:


    - #### getConfidences

      ```
      public static double[] getConfidences(mulan.classifier.MultiLabelLearner trainedLearner,
                            IdInstance instance)
      ```

      // input: trained model // actions: get MultiLabelOutput from trained
      model using // .makePredictions(Instance), // get confidence[] --> one
      confidence for each class label

      Parameters:
      :   `trainedLearner` -
      :   `instance` -

      Returns:


    - #### getUncertaintyValue

      ```
      public static double getUncertaintyValue(double confidence)
      ```

      // the confidence certainty decreases the closer it is to 0.5 // (e.g.
      for confidence=0.9, confidence\_certainty = 0.4 but for //
      confidence=0.55, confidence\_certainty = 0.05) // in short, the closer to
      50% the confidence is, the most distant the // prediction is from being
      certainly false (0%) // or certainly true (100%) //
      Confidence\_uncertainty = 0.5 - | (0.5 - average confidence) |

      Parameters:
      :   `a` - confidence between 0.0 and 1.0

      Returns:


- Overview
- Package
- Class
- Use
- Tree
- Deprecated
- Index
- Help

- Prev Class
- Next Class

- Frames
- No Frames

- All Classes

- Summary:
- Nested |
- Field |
- Constr |
- Method

- Detail:
- Field |
- Constr |
- Method
